# Autoantibodies Against the Immunodominant Bullous Pemphigoid Epitopes Are Rare in Patients With Dermatitis Herpetiformis and Coeliac Disease

**DOI:** 10.3389/fimmu.2020.575805

**Published:** 2020-09-25

**Authors:** Antti Nätynki, Jussi Tuusa, Kaisa Hervonen, Katri Kaukinen, Outi Lindgren, Laura Huilaja, Nina Kokkonen, Teea Salmi, Kaisa Tasanen

**Affiliations:** ^1^PEDEGO Research Unit, Department of Dermatology, Medical Research Center Oulu, Oulu University Hospital, University of Oulu, Oulu, Finland; ^2^Department of Dermatology, Tampere University Hospital, Tampere, Finland; ^3^Celiac Disease Research Center, Faculty of Medicine and Health Technology, Tampere University, Tampere, Finland; ^4^Department of Internal Medicine, Tampere University Hospital, Tampere, Finland; ^5^Department of Pathology, Medical Research Center Oulu, Oulu University Hospital, University of Oulu, Oulu, Finland

**Keywords:** bullous pemphigoid, dermatitis herpetiformis, coeliac disease, epitope analysis, blistering skin disease

## Abstract

Dermatitis herpetiformis (DH) is an extraintestinal manifestation of coeliac disease (CD). Patients with DH have an elevated risk of development of another autoimmune blistering skin disease, bullous pemphigoid (BP). In this study we investigated whether patients with DH and CD (mean age for both 49 years) have circulating autoantibodies against BP180, the major BP autoantigen. ELISA tests showed that only a few DH (3/46) and CD (2/43) patients had BP180-NC16A IgG autoantibodies. Immunoblotting found that more than half of the DH samples contained IgG autoantibodies against full-length BP180. Epitope mapping with 13 fusion proteins covering the BP180 polypeptide revealed that in DH and CD patients, IgG autoantibodies did not target the NC16A or other epitopes typical of BP but recognized other intracellular and mid-extracellular regions of BP180. None of the analyzed DH and CD patients with either ELISA or immunoblotting positivity had IgG or IgA reactivity against the cutaneous basement membrane in indirect immunofluorescence analysis or skin symptoms characteristic of BP. Although only a minority of middle-aged DH patients had IgG autoantibodies against the immunodominant epitopes of BP180, our results do not exclude the possibility that intermolecular epitope spreading could explain the switch from DH to BP in elderly patients.

## Introduction

Bullous pemphigoid (BP) and dermatitis herpetiformis (DH) are both autoimmune subepidermal blistering skin diseases. Intense pruritus is present in both diseases, but tense bullae are typical for BP, whereas symmetrically distributed small blisters, papules and excoriations in elbows, knees and buttocks are common in DH (1, 2). BP typically affects people aged 75–80 years (1), while DH patients are generally younger, its incidence being highest in individuals aged 50–69 years (2). The incidence of BP has recently increased in several countries (3) and that of DH decreased (4). Recent epidemiological work revealed a special connection between these two autoimmune diseases: a prior diagnosis of DH was found to increase the risk of BP by 22-fold (5).

Autoantibodies against cutaneous proteins play a central role in the pathogenesis of DH and BP, but the factors that lead to the breakage of immunotolerance and disease onset are still largely unknown (1). DH is a cutaneous manifestation of coeliac disease (CD) and both CD and DH are characterized by the targeting of enzymes of the transglutaminase family by IgA autoantibodies (2). Proteolytic gliadin peptides from ingested gluten induce production of IgA class autoantibodies against both native and gliadin-associated tissue transglutaminase 2 (TG2) (2). These antibodies are detectable in the serum and small bowel mucosa of CD patients, but in only about 75% of DH patients (6, 7). Typically the sera of DH patients contain anti-epidermal transglutaminase (TG3) IgA autoantibodies, which show up on direct immunofluorescence microscopy (DIF) analysis of the skin as granular IgA deposits in the papillary dermis (8, 9). ELISA-based TG2 tests are widely used and are highly accurate for the diagnosis of CD, but a negative result does not exclude DH (10). TG3 ELISA tests are also available, but are currently used only for research purposes (2). A lifelong gluten-free diet is the basis of treatment of both CD and DH (6).

The major autoantigen in BP is BP180, which is one of the structural proteins required to adhere basal keratinocytes to the cutaneous basement membrane (11). The subepidermal blistering characteristic of BP is caused by IgG class autoantibodies binding to BP180, thereby interfering with hemidesmosome function. As with DH, the golden standard technique for BP diagnosis is DIF analysis, which reveals linear IgG and complement 3 (C3) staining in the cutaneous basement membrane zone. The immunodominant epitope of BP180 is the NC16A domain and BP180-NC16A IgG autoantibodies are commonly measured using ELISA tests. IgA autoantibodies against 120 and 97 kDa ectodomain fragments of BP180 cause linear IgA bullous dermatosis (LABD), another disease of the pemphigoid group (11, 12).

In the present study we investigated the prevalence of transglutaminase and BP180 autoantibodies in patients with DH, CD, and BP and in healthy controls. We also performed a detailed analysis of which epitopes of BP180 the autoantibodies of patients with CD and DH recognize and whether these autoantibodies are able to bind to the cutaneous basement membrane.

## Materials and Methods

### Patients

The research ethics committees of Tampere University Hospital and the Northern Ostrobothnia Hospital District, Finland approved the prospective collection of human DH, CD, BP, and control sera. Additional control samples were obtained from the Northern Finland Biobank Borealis, Oulu, Finland (https://www.ppshp.fi/Tutkimus-ja-opetus/Biopankki/Pages/Biobank-Borealis-briefly-in-English.aspx). All samples were taken after written informed consent. The blood samples were obtained from DH (*n* = 46) and CD (*n* = 43) patients diagnosed as described previously (10) in the Department of Dermatology and the Department of Internal Medicine, Tampere University Hospital. The samples of 22 of the 46 DH patients were taken at the time of DH diagnosis; five were taken 1 year after DH diagnosis while patients were receiving a gluten-free diet. The remaining 19 DH samples were obtained from patients several years after their diagnoses (mean time 23 years) and had been challenged with gluten for 6 months (13). The samples of 22 of the 43 CD patients were obtained at the time of diagnosis and the remaining 21 during a gluten-free diet 1 year after CD diagnosis. BP patients (*n* = 34) were diagnosed as described previously (14) in the Department of Dermatology, Oulu University Hospital and all samples were taken at the time of diagnosis. None of the BP patient had preceding DH or CD. In patients with DH, CD, BP and controls, respectively, the mean ages ± SD in years were 48.7±15.1, 48.8±13.4, 79.9±7.9, and 49.4±4.6. The number of females/total and proportion of females were 16/46 (34.8%); 28/43 (65.1%); 14/34, (41.2%), and 31/48 (64.6%), respectively. All patients were of Caucasian origin.

### ELISA Assays

The QuantaLite® h-tTG IgA ELISA (Inova Diagnostics Inc., San Diego, CA, USA) was used to identify TG2 IgA antibodies. Values ≥ 20 units were considered to be positive. The anti-heTG IgA ELISA (Immundiagnostik AG, Bensheim, Germany) was used to analyze TG3 IgA antibodies. Values > 22.0 U/ml were considered to be positive. The MESACUP BP180 ELISA (Medical and Biological Laboratories Co., Ltd., Nagoya, Japan), was employed to identify BP180-NC16A IgG autoantibodies, with a positivity cut-off value of ≥ 9 U/ml. All the ELISA assays were performed according to manufacturer's instructions.

### Immunoblotting and Epitope Mapping

For immunoblotting against full-length and plasmin digested human BP180 COS-7 cells were transfected with human BP180 cDNA (15) using Lipofectamine 3000 (Life technologies, Carslbad, CA, USA). Culture medium [[DMEM, Life technologies] with 10% fetal bovine serum and penicillin-streptomycin] was supplied with 50 μg/ml ascorbic acid. Forty eight hours after transfection cells were harvested. Whole cell extracts were prepared as described earlier (16).

To prepare plasmin digests, cell membranes from COS-7 cells transiently transfected with BP180 cDNA were prepared by suspending scraped cells into a lysis buffer [20 mM Tris-Cl pH 8.0, 50 mM NaCl, 1 × Complete protease inhibitor cocktail [Roche-diagnostics GmbH, Mannheim, Germany]]. After incubation in ice for 15 min, the cells were lysed by three repetitions of a freezing-melting cycle. After centrifugation at 10 000 × g for 1 h, the supernatant was discarded and the pellet was resuspended into a digestion buffer (50 mM Tris-Cl, pH 8.9, 150 mM NaCl). Digestion was done by 2 U/ml plasmin (Roche-diagnostics GmbH, Mannheim, Germany) added into a membrane fraction and incubated at 37°C for 3 h. An equal volume of 2X Laemmli SDS-sample buffer was added immediately after plasmin incubation.

In epitope mapping 50 ng of each GST-BP180 fusion protein expressed in *E. coli* (17) were used as antigen. Immunoblotting was performed as described earlier (16, 17) with a modification that serum samples were diluted in 1:100 in 3% BSA-TBST and horseradish peroxidase (HRP)- conjugated goat anti-human IgG (Sigma-Aldrich, St. Louis, MO, USA) or HRP-conjugated goat anti-human IgA (Life technologies) were used as secondary antibodies. Anti-BP180 (Abcam, Cambridge, UK), anti-NC16A (18) and anti-GST (Thermo Fisher Scientific, Inc., Waltham, MA, USA) antibodies with HRP-conjugated goat anti-rabbit IgG (Sigma-Aldrich) or anti-mouse IgG (Abliance, Compiègne, France) were used to detect the specific protein bands. Protein bands were visualized with ECL Prime substrates (GE Healthcare, Buckinghamshire, UK) and LAS Imager 3000 (Fujifilm, Tokyo, Japan).

### Indirect Immunofluorescence (IIF)

IIF was performed on the serum samples that were tested positive in the ELISA assays and/or detected the 180 kDa band in immunoblotting. To prepare salt-split skin samples the fresh human skin biopsy was incubated for 20 h in salt solution (1 M NaCl, 2 mM EDTA, 200 mM Tris, pH 7.4) at room temperature to separate the epidermis from the dermis before freezing and sectioning. The frozen normal or salt-split human skin sections were then incubated overnight with serum samples diluted in 1% BSA-PBS over a range of concentrations from 1:100 to 1:10. FITC-conjugated anti-human IgG (DAKO, Glostrup, Denmark) or anti-human IgA (Invitrogen, Waltham, MA, USA) were used as secondary antibodies. Blinded evaluation of cryosections was performed by three independent observers.

### Data Analysis

Statistical analyses were conducted using the IBM SPSS software (IBM, Armonk, NY, USA). Differences between groups in ELISA results (continuous, non-normal variable) were analyzed using the Kruskal-Wallis test. Mean and median values and percentiles were reported, as appropriate. In epitope mapping, antibody-recognized bands were densitometrically analyzed using the ImageJ software package (NIH, Bethesda, MD, USA). In densitometric analysis, the background was determined from the empty region in the lane above the band. The bands were classified by two independent researchers with an ordinal scale: “0” = no band, “1” = weak, “2” = strong, “3” = very strong (saturated). These subjective categorizations were confirmed by examination of the densitometric value of each band, relative to the others in the same blot. Differences between groups in epitope mapping were compared pairwise using the Fisher's exact test. A two-tailed *P-*value of 0.05 or less was considered to be statistically significant.

## Results

### TG2 and TG3 IgA Autoantibody Profiles

**Figure 1:**
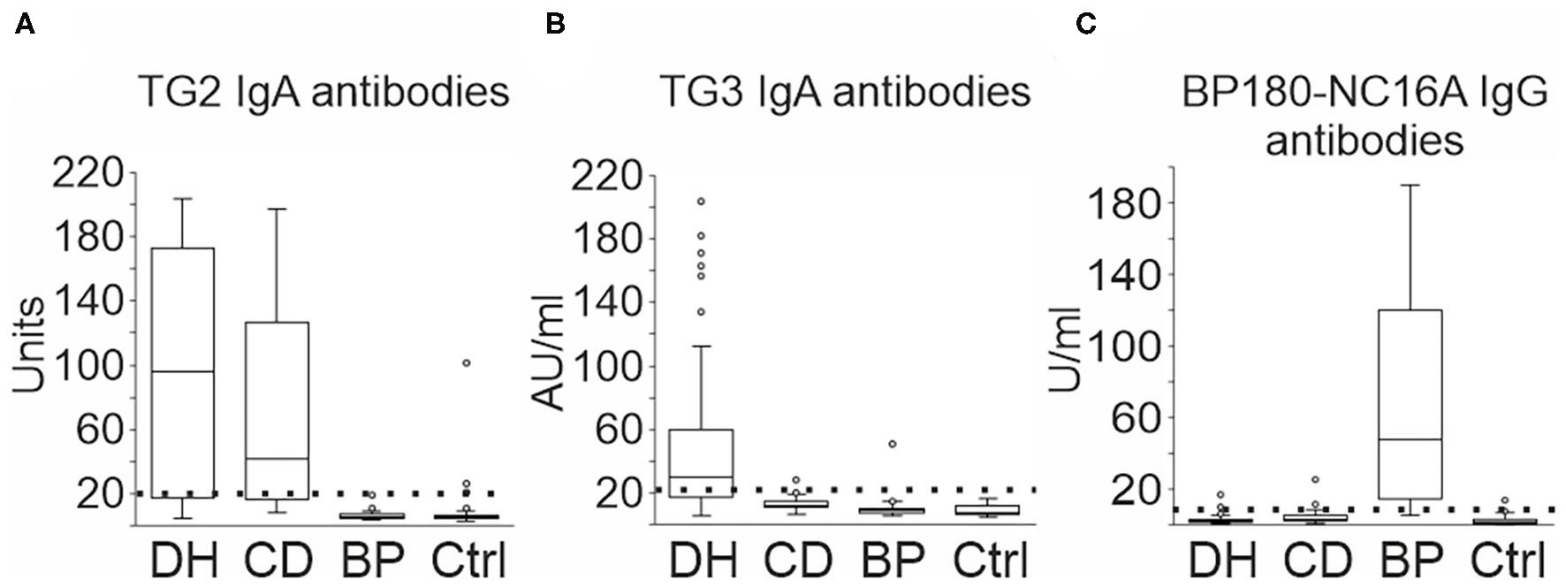
TG2, TG3, and BP180-NC16A autoantibodies in DH, CD, and BP serum samples. 46 DH, 43 CD and 34 BP, and 48 control sera were tested for IgA or IgG reactivity against TG2, TG3, and BP180-NC16A using ELISA. **(A)** Increased TG2 IgA autoantibodies were detected in 33 DH samples, 31 CD samples, and 3 controls. **(B)** Increased TG3 IgA autoantibodies were detected in 29 DH samples, one CD, and one BP sample. **(C)** Increased BP180-NC16A IgG autoantibodies were detected in 28 BP, three DH, two CD, and two control samples. The boxplot represents the distribution of values in varying units as given by the manufacturer. Boxes depict the 25 and 75th percentiles (interquartile range, IQR) and line in the box is median. Whiskers indicate last values within 1.5 times the IQR from the median, and outliers are marked with circles. BP, bullous pemphigoid; CD, coeliac disease; Ctrl, control; DH, dermatitis herpetiformis.

We first analyzed the incidence of TG2 and TG3 IgA autoantibodies in the sera of patients with DH (*n* = 46), CD (*n* = 43) and BP (*n* = 34), and healthy controls (*n* = 48) using ELISA tests. TG2 IgA autoantibodies were detected frequently in the DH (33/46, 71.7%) and CD (31/43, 72.1%) groups, but in none of the BP and only in a few of the control samples (3/48, 6.3%) (Figure 1A). High prevalence of TG3 autoantibodies clearly differed DH group from others: A level of IgA-autoantibodies above the cut-off value was found in 29/46 (63.0%) of DH samples, 1/43 (2.3%) of CD samples, and none of controls (Figure 1B). One of the 34 BP samples (2.9%) was positive (Figure 1B).

### BP180-NC16A IgG Autoantibody Profiles

The majority of BP group sera (28/34, 82.4%) were classified as positive for BP180-NC16A IgG antibodies (Figure 1C). There were 3/46 (6.5%) positive sera in the DH group, 2/43 (4.7%) in the CD group and 2/48 (4.2%) in the control group (Figure 1C). None of the DH and CD patients with positive BP180-NC16A ELISA values were following a gluten-free diet at the time when the serum samples were drawn. In the BP group, 32% of the values were over 100 U/ml (median 47.5 U/ml), whereas in the other groups positive values ranged from 9.4 to 25.1 U/ml (Figure 1C). All three positive DH samples were also positive for TG2 and TG3 autoantibodies. Both the positive CD samples were also positive for TG2 autoantibodies, but TG3 negative. Of the two BP180-NC16A ELISA positive control samples, one serum was positive for TG2 autoantibodies and the other was negative for both TG2 and TG3 autoantibodies. None of the patients whose sera were positive for BP180-NC16A autoantibodies exhibited BP-like symptoms.

### Immunoblotting Analyses of Autoantibodies Against BP180

**Figure 2:**
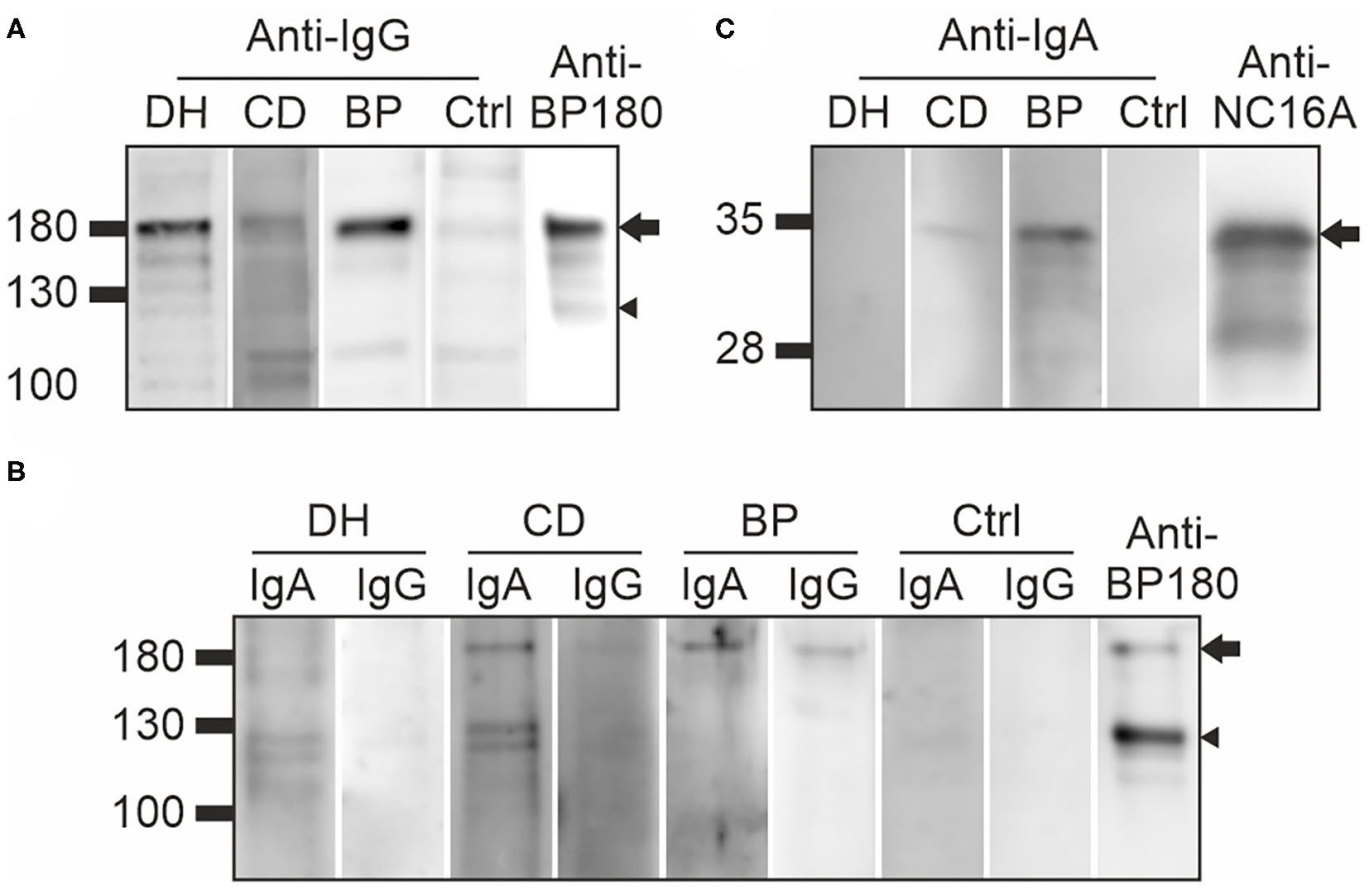
Immunoblotting of DH, CD, and BP sera against full-length and plasmin-digested recombinant BP180, and the recombinant NC16A domain **(A)** Examples of DH, CD, BP, and control sera IgG autoantibodies that recognized 180 kDa BP180 (arrow) and some also the 120 kDa ectodomain (arrowhead). **(B)** Examples of DH, CD, BP, and control sera containing IgA and/or IgG autoantibodies that recognize 180 kDa BP180 (arrow) or the 120 kDa ectodomain (arrowhead) after partial plasmin digestion. Specific anti-BP180 antibody was used as a positive control. **(C)** The recombinant BP180-NC16A domain of 36 kDa was recognized by IgA autoantibodies in the CD and BP samples (arrow), but not the DH and control samples. NC16A-BP180-specific IgG antibody was used as a positive control. BP, bullous pemphigoid; CD, coeliac disease; Ctrl, control; DH, dermatitis herpetiformis.

Immunoblotting for IgG autoantibodies against full-length BP180 was carried out in 20 samples from the DH group, 20 from the CD group, three from the BP group and 21 controls. These samples included all those that had tested positive for BP180-NC16A autoantibodies in the ELISA, plus random negative samples from the DH, CD, and control groups. All the tested BP sera recognized the full-length recombinant BP180 with a strong intensity (Figure 2A, Table 1). Of the analyzed DH sera, 55% contained IgG autoantibodies that recognized the 180 kDa band corresponding to full-length BP180 (Table 1). The proportions of BP180-recognizing sera in the CD and control groups were lower, at 40% and 38%, respectively (Table 1).

**Table 1:** Immunoblotting of DH, CD, BP, and control sera against full-length and plasmin-digested BP180, and the BP180-NC16A domain.

**Detected polypeptide in immunoblotting**	**DH**	**CD**	**BP**	**Controls**
	+/total (%)	+/total (%)	+/total (%)	+/total (%)
Recombinant full-length BP180, anti-IgG^a^	11/20 (55.0)	8/20 (40.0)	3/3 (100.0)	8/21 (38.1)
BP180 after plasmin digestion, anti-IgA^b^	1/20 (5.0)	2/20 (10.0)	2/20 (10.0)	3/22 (13.6)
The 120-kDa ectodomain after plasmin digestion, anti-IgA^b^	5/20 (25.0)	5/20 (25.0)	6/20 (30.0)	3/22 (13.6)
BP180 after plasmin digestion, anti-IgG^c^	0/6	2/6	4/8	0/5
The 120-kDa ectodomain after plasmin digestion, anti-IgG^c^	1/6	0/6	3/8	0/5
BP180-NC16A domain anti-IgA^d^	0/20 (0.0)	1/20 (5.0)	4/20 (20.0)	0/21 (0.0)
BP180-NC16A domain anti-IgG^d^	0/46 (0.0)	0/43 (0.0)	15/23 (65.2)	0/21 (0.0)

*BP, bullous pemphigoid; CD, coeliac disease; DH, dermatitis herpetiformis*.

a *Sera were analyzed by immunoblotting using human recombinant full-length BP180 expressed in COS-7 cells as a target antigen. Three BP sera were used as positive controls to detect full-length BP180*.

b *Sera were analyzed by immunoblotting with anti-IgA secondary antibody using partially plasmin-digested human recombinant full-length BP180 expressed in COS-7 cells as a target antigen*.

c *Sera which had reactivity against the full-length BP180 and/or the 120 kDa fragment after plasmin digestion were analyzed by immunoblotting with anti-IgG secondary antibody using partially plasmin-digested human recombinant full-length BP180 expressed in COS-7 cells as a target antigen*.

d *Sera were analyzed by immunoblotting using glutathione transferase-BP180 fusion protein FP5 corresponding the human immunodominant NC16A domain expressed in E. coli as a target antigen*.

Previous reports that LABD IgA autoantibodies target the shed ectodomain and 97 kDa fragment of BP180 particularly strongly (11, 19, 20) prompted us to investigate whether plasmin digestion of BP180 would produce neoepitopes that would be recognized by IgA autoantibodies in samples from DH (*n* = 20), CD (*n* = 20), and BP (*n* = 20) patients and healthy controls (*n* = 22). After partial plasmin digestion, the remaining undigested full-length BP180 was recognized by 5% of DH samples, 10% of both CD and BP samples and 13.6% of control samples (Figure 2B, Table 1). The 120 kDa plasmin-digested fragment was recognized by IgA autoantibodies in 25% of the DH, 25% of the CD and 30% of the BP samples, but reactivity was also detected in 13.6% of the control samples (Figure 2B, Table 1). The DH (*n* = 6), CD (*n* = 6), BP (*n* = 8), and control (*n* = 5) sample with IgA reactivity against the plasmin-treated BP180 were further tested for IgG reactivity. Of these, one DH samples had both IgA and IgG autoantibodies against the 120 kDa plasmin-digested fragment and two CD samples had IgA and IgG reactivity against the remaining undigested full-length BP180 (Table 1, Figure 2). Nearly half of the BP samples had both IgA and IgG reactivities against the plasmin treated BP180 (Table 1, Figure 2).

The presence of IgA autoantibodies targeting the main immunodominant NC16A domain of BP180 was analyzed with immunoblotting using the previously produced GST fusion protein corresponding to this region (FP5; amino acids (aa) 489–567) (17). This analysis found 20% of the BP samples to be positive, but outside this group, only one sample (from the CD group) contained IgA antibodies against the NC16A domain (Figure 2C, Table 1).

### IgG Autoantibodies Target Different Epitopes of BP180 in DH and CD Than in BP

**Figure 3:**
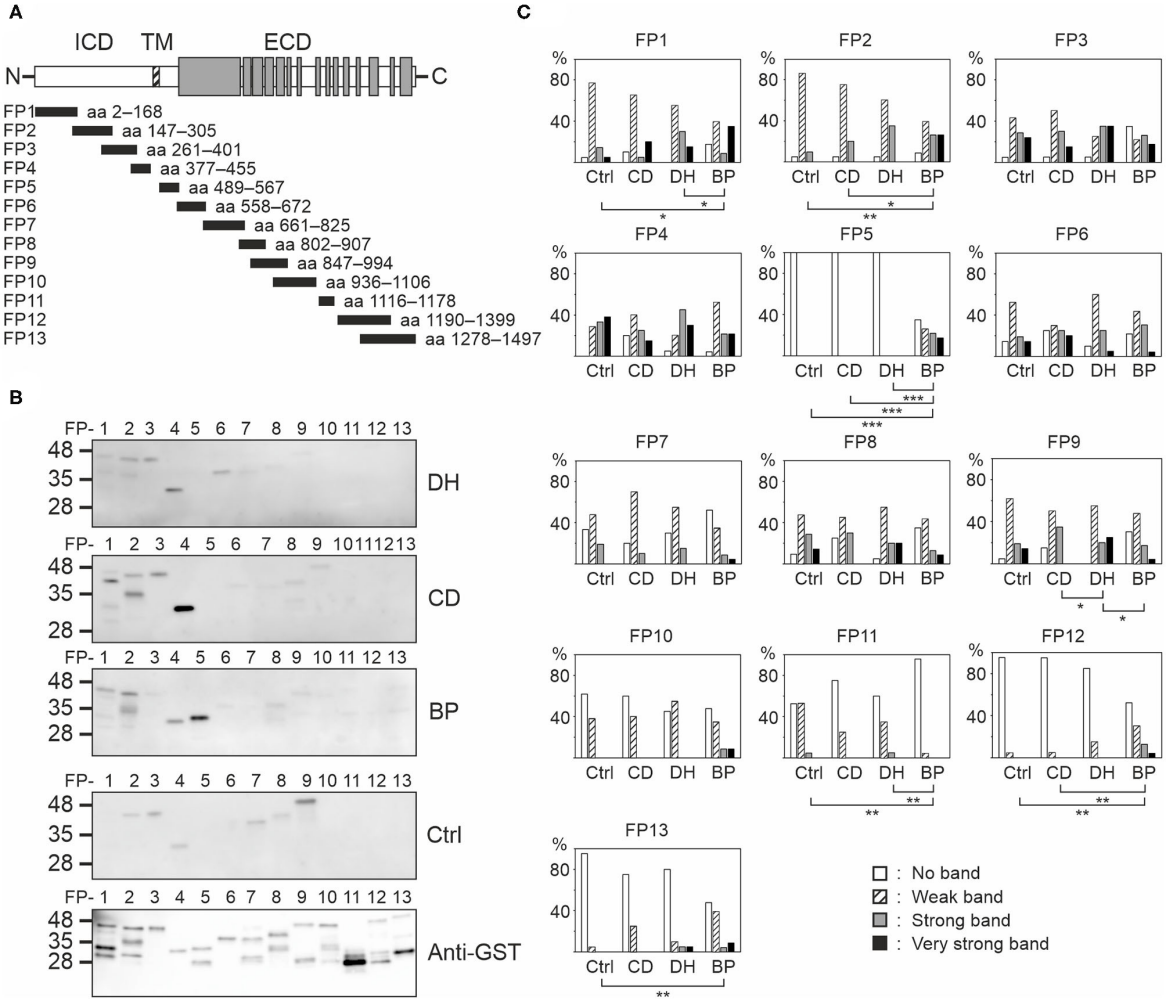
Mapping of linear BP180 epitopes reveals differences between DH, CD, BP, and control sera. **(A)** Equal amounts of glutathione-S-transferase (GST)-BP180 fusion proteins were immunoblotted with 20 DH, 20 CD, 23 BP, and 21 control serum samples and anti-human IgG secondary antibody. The gray areas depict collagenous domains. **(B)** Representative examples from each group are shown. **(C)** Relative frequencies of densitometrically classified immunoblotting signal intensities for indicated fusion proteins are shown. Statistically significant differences between groups are marked with asterisks, where two-sided *P*-values are: *P ≤ 0.05, **P ≤ 0.01, ***P ≤ 0.001. BP, bullous pemphigoid; CD, coeliac disease; Ctrl, control; DH, dermatitis herpetiformis; ECD, extracellular domain; FP, fusion protein; ICD, intracellular domain; TM, transmembrane domain.

To determine which BP180 epitopes are recognized by DH and CD sera epitope mapping using 13 GST-fusion proteins covering most of the BP180 polypeptide (Figure 3A) was carried out in 20 samples from the DH group, 20 from the CD group, 23 from the BP group and 21 from the control group. DH, CD, and control groups were the same samples that were used in immunoblotting against full-length BP180. BP samples were the same as previously characterized (17). To analyze immunoreactivity against the major immunodominant epitope in BP more accurately, immunoblotting against the NC16A domain-containing fusion protein FP5 was carried out in all DH (*n* = 46) and CD (*n* = 43) samples that were used for ELISA measurements (Table 1).

Each serum from all groups recognized several fusion proteins (Figures 3B,C, Supplementary Table 1). Although the numbers and intensities of recognized fusion proteins varied between individual sera, clear differences between groups emerged (Figure 3C, Supplementary Tables 2, 3). Only IgG autoantibodies present in BP sera bound with NC16A containing fusion protein FP5. Of the tested BP samples, 65.2% were positive for FP5, and the majority of these had also tested positive for BP180-NC16A autoantibodies in ELISA. Carboxyterminal regions known to harbor epitopes targeted by autoantibodies of some BP patients (21–23) were recognized more frequently by BP samples than by those of other groups as were the intracellular epitopes mapping to FP2 (Figure 3C). In contrast FP11 (aa 1116–1178) was less often recognized by BP samples than by those in the other groups (Figure 3C). Samples from the DH group differed significantly from those of the CD and BP groups in terms of recognition of FP1 (aa 2–168) and FP9 (aa 847–994), respectively (Figure 3C). Recognition of FP3, FP4, FP6, FP7, FP8, and FP10 did not vary statistically between the study groups (Figure 3C).

### DH and CD Patients Have No Circulating IgG or IgA Autoantibodies Against the Cutaneous Basement Membrane

**Figure 4:**
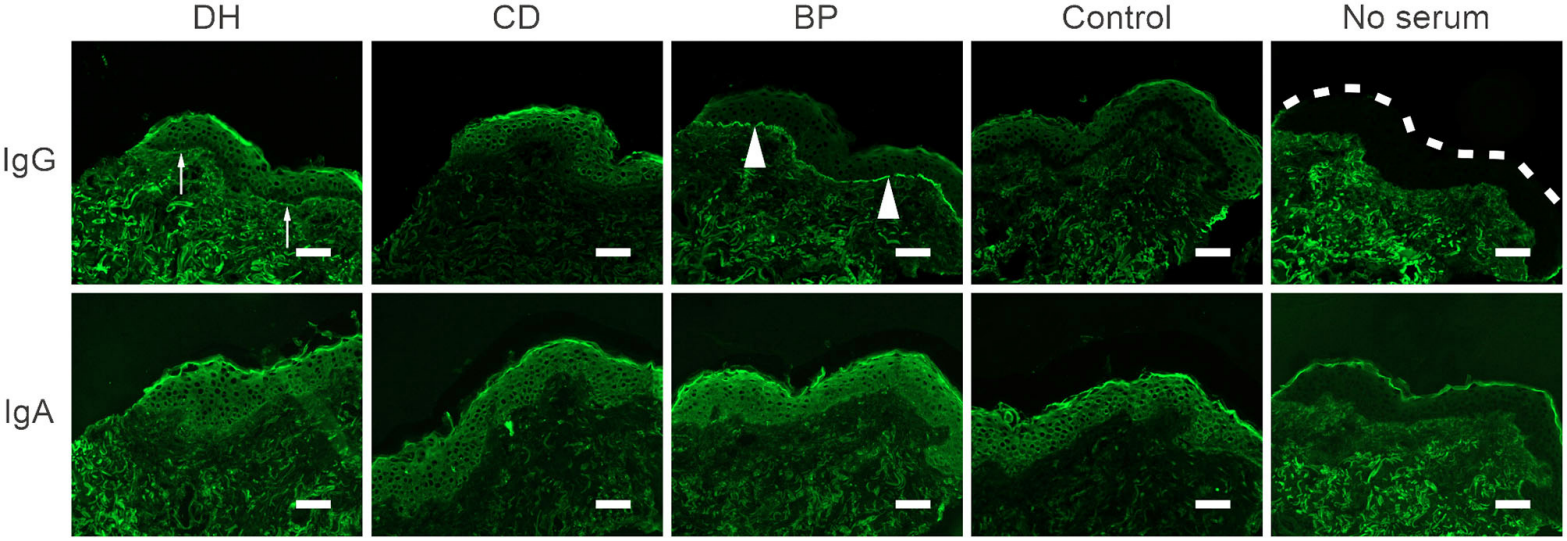
Autoantibodies in DH, CD and control sera do not react with the cutaneous basement membrane zone. Representative images of indirect immunofluorescence (IIF) analysis in frozen human skin sections. 13 DH, 9 CD, and 9 control sera were tested with anti-human IgG-FITC or IgA-FITC as a secondary antibody. BP serum served as a positive control. IgG-autoantibodies in BP sera bound linearly to the basement membrane zone of human skin (arrowheads) and two DH and two CD sera showed faint positive depositions (arrows). All IIF analysis with anti-IgA were negative. Scalebar = 50 μm, dashed line marks the epidermal edge. BP, bullous pemphigoid; CD, coeliac disease; Ctrl, control; DH, dermatitis herpetiformis.

Indirect immunofluorescence (IIF) analysis with IgG as the secondary antibody was performed for 13 samples from the DH group, nine from the CD group, five from the BP group and nine from the control group. All samples had previously tested positive for BP180-NC16A autoantibodies in ELISA, full-length BP180 or both (Table 1). Two of the 13 DH sera and two of the nine CD sera showed a faint positive IgG immunofluorescence signal at the cutaneous basement membrane, whereas a clear linear IgG positivity was detected in all five BP sera (Figure 4). All healthy control sera remained negative. Serum samples from patients who returned a faint positive signal for IgG in the IIF analyses were analyzed using the salt split skin technique. All were negative (data not shown). In addition, IIF with IgA as the secondary antibody was performed for DH, CD, BP, and control sera that had tested positive for TG3, BP180-NC16A, or both. All samples were negative for IgA (Figure 4).

## Discussion

Numerous studies have demonstrated that the risk of developing BP is significantly elevated in individuals who have neurodegenerative diseases or use dipeptidyl peptidase-4 inhibitors (gliptins) to treat diabetes (24–27). A preceding diagnosis of another itching and blistering disease, DH, is associated with an even greater risk elevation (5). The molecular mechanisms by which different risk factors induce the immune system to act against BP180 are unknown, although neuroinflammation leading to the failure of self-tolerance against BP180, immunological changes caused by gliptins and epitope spreading have all been suggested (16, 17, 27, 28). In epitope spreading the initial B and/or T-cell response expands from the primary to one or more secondary epitopes as the autoimmune disease progresses (28, 29). In intramolecular epitope spreading, there is a shift in the epitopes that respond to the same antigen, whereas in intermolecular epitope spreading autoantibodies are raised against different antigens. Some patients with dementia, multiple sclerosis (MS) or Parkinson's disease have non-pathogenic anti-BP180 autoantibodies that target epitopes outside the immunodominant NC16A domain (17, 30). The significance of these non-pathogenic autoantibodies for the development of BP is currently poorly understood, but it can be speculated that over time some individuals may develop BP180-NC16A autoantibodies through intramolecular epitope spreading.

The aim of our current work was to investigate whether intermolecular epitope spreading may explain why individuals with gluten-induced autoimmunity carry an elevated risk for BP. This idea was inspired by a current hypothesis that DH develops as an intermolecular epitope spreading phenomenon against TG3 following a sub-clinical or neglected occurrence of CD (2, 31). However, the presence of BP180-NC16A IgG autoantibodies was specific for patients with BP: only a minority of CD (4.7%) and DH (6.5%) samples were positive in BP180-NC16A IgG tests, and the ELISA values in these samples were clearly lower than those of the BP samples. Thus, elevated BP180-NC16A IgG autoantibodies were found among DH patients approximately as frequently as reported previously for elderly patients with pruritic disorders (32, 33). The rate of BP180-NC16A IgG ELISA positivity of our control samples (4.2%) was similar to that reported previously for healthy controls or patients affected by immediate-type allergic disorders (32, 33). Surprisingly high rates of increased BP180-NC16A ELISA values were recently reported in patients with non-BP skin diseases: the portion of increased BP180 IgG autoantibodies varied between 4.3% in patients with erythema multiforme and 44.4% in patients with Stevens-Johnson syndrome (34). These findings were probably driven by inflammation associated with the breakage of the cutaneous basement membrane zone. We conclude that the incidence of BP180-NC16A pathogenic autoantibodies among DH and CD patients is at a similar level to that of healthy controls and these results do not support the hypothesis of intermolecular epitope spreading from DH and CD to BP.

We found that elevated levels of BP180-NC16A autoantibodies were mostly detected in BP patients. Similarly, high TG2 and TG3 ELISA values were mostly present in patients with CD and DH, and only a few healthy control samples were positive for TG2 IgA autoantibodies. Serological evidence of co-existing gluten-induced autoimmunity was scarce in BP patients: all BP samples (*n* = 34) were negative for TG2 and only one BP sample had increased IgA reactivity for TG3. A slightly higher incidence of elevated TG2 and TG3 IgA autoantibodies was recently detected in patients with another disease of the pemphigoid group, the pregnancy-associated pemphigoid gestationis (35). Three of 12 patients with pemphigoid gestationis had an elevated level of IgA TG2 autoantibodies and one had a high IgA TG3 autoantibody level (35). Anti-TG3 IgA antibodies have also been found in 3% of patients with atopic dermatitis (*n* = 300), but the significance of this finding is not known (36). Taken together, our current results based on the commercially available BP180, TG2, and TG3 ELISA tests do not support the idea that epitope spreading explains the evolution from DH or CD into BP.

Although both DH and CD are more common in Finland than almost anywhere else in the world (4), we were not able to obtain samples from BP patients with preceding DH or CD. There has been only one case report of the concomitant presence of autoantibodies against BP180 and TG2, in a 77-year-old patient who was diagnosed with DH and BP simultaneously (37). In our current study positive sera were very rare in the DH, CD, and control groups, being present in <7% in each group. This result may have been related to patient demographics. The mean age of DH patients was on average 20 years lower than that recently reported in patients with a BP diagnosis preceded by DH (68.8 years) (5). Gluten-induced autoimmunity has a much earlier onset than BP, and in Finland patients with DH or CD have a high level of adherence to the recommended gluten-free diet (38). Combined, these factors may have skewed our DH and CD patient population away from elderly individuals, which in turn may have influenced the incidence of BP180 autoantibodies in our current study. In previous reports the majority of patients with DH followed by BP were over 70 years old, the age varying between 48 and 84 years (28, 37). The effect of age on the occurrence of BP180 autoantibodies is supported by our previous results: the incidence of elevated BP180-NC16A autoantibody levels was higher (18%) in patients with Alzheimer's disease (AD) (mean age 72 years) than those with MS (7.7%, mean age 49.4 years) (16, 17), although, compared with various forms of dementia, MS is associated with a clearly higher risk for developing BP (24). Previous IIF studies have shown that few serum samples of AD or MS patients are able to bind to the cutaneous basement membrane zone (16, 17, 30). Similarly, our DH and CD samples with positivity against BP180 remained negative using the salt-split skin technique. We propose that these positive sera bind either to cryptic BP180 epitopes in immunoblotting or a folded conformer of an isolated NC16A domain in ELISA, which are not recognized in the natively folded trimeric full-length BP180 in the skin (17).

In immunoblotting, IgG reactivity against the full-length BP180 was detected in more than half of the DH samples. This is a similar proportion to that found in patients with MS, who have the highest risk for BP among neurological patients (17, 24). IgG autoantibodies recognizing full-length BP180 were also detected in patients with CD and healthy controls, a finding that drove our decision to perform anti-BP180 IgG linear epitope mapping. We found that the levels of epitope recognition of anti-BP180 IgG autoantibodies among the sera of DH and CD patients were similar to those we have previously reported in patients with MS and AD (17). All these groups, as well as healthy controls, harbor apparently non-pathogenic autoantibodies against the BP180 intracellular domain and the mid-portion of the ectodomain with a wide degree of inter-individual variation in the number of GST-BP180 fusion proteins recognized, and in the intensity of recognition. Our DH and CD sera had a slightly higher immunoreactivity against the intracellular FPs FP1 and FP2 than the BP sera, and lower reactivity against the mid-ectodomain FPs FP9 and FP11. This finding could be due to differences in antigen presentation of cryptic epitopes in the skin and intestinal mucosa. Currently it is not known whether and how the presence of these non-pathogenic autoantibodies might explain the elevated risk of BP in these patients. One possibility is that after intermolecular epitope spreading from TG2/3 to BP180, intramolecular epitope spreading events could induce the development of pathogenic autoantibodies. However, as seen in MS and AD patients, IgG antibodies in the DH, CD, and control sera never recognized the fusion protein 5 (FP5) that corresponds to the BP immunodominant NC16A domain, and only rarely recognized the carboxyterminal ectodomain (17). A single patient with CD (age 63 years) had IgA autoantibodies that recognized the immunodominant NC16A domain, but the BP180-NC16A IgG ELISA was negative in this patient, and skin symptoms typical of BP were not present. It is noteworthy that in the immunoblotting experiments, the full-length BP180 and fusion proteins used for epitope mapping were recognized not only by DH and CD sera, but also by control samples, and this should be considered when interpreting our results.

It is not fully understood why in LABD the 120 and 97 kDa fragments of BP180 are targeted by IgA rather than IgG autoantibodies (11, 12). Also, BP patients have IgA autoantibodies that recognize the aminoterminal epitopes of the BP180 ectodomain particularly strongly (39). Relatively few of our DH, CD and BP sera contained IgA autoantibodies against full-length BP180, but, interestingly, 25–30% did display IgA reactivity against the 120 kDa band generated by plasmin digestion of recombinant BP180. However, only a few DH and CD samples had simultaneous IgG and IgA reactivity against the plasmin treated BP180. The ability of IgA antibodies against the 97 kDa LABD to induce dermal-epidermal separation has been demonstrated in a passive transfer mouse model (40), but the significance and role of IgA autoantibodies in the pathogenesis of BP is unknown.

The hypothesis of intermolecular epitope spreading from non-BP to BP type autoimmunity suggests that autoimmune diseases such as MS, DH, and CD cause tissue destruction, which allows the exposure of cryptic neoepitopes that are normally hidden from the immune system. It also suggests that the loss of tolerance against the secondary target like BP180, either by targeting of the immunodominant NC16A domain or other BP180 regions is followed by intramolecular epitope spreading to non-NC16A and NC16A epitopes, respectively. Our current data do not support the transition of DH and CD to BP by epitope spreading, because in this study sample only a few of middle-aged DH and CD patients were found to carry BP180-NC16A autoantibodies that did not recognize the skin basement membrane zone in IIF analysis, and accordingly, were unable to cause clinical signs of BP. A relatively large proportion of the DH and CD sera also contained non-pathogenic autoantibodies with reactivity to linear epitopes outside the immunodominant NC16A and carboxyterminal domains, but the role played by these autoantibodies in the shift from DH or CD to BP is unclear. However, our current data do not fully exclude the possibility that intermolecular epitope spreading from TG2 or TG3 to BP180 could play a role in the transition from DH or CD to BP among elderly patients.

## Data Availability Statement

The raw data supporting the conclusions of this article will be made available by the authors, without undue reservation.

## Ethics Statement

The studies involving human participants were reviewed and approved by the research ethics committees of Tampere University Hospital and Northern Ostrobothnia Hospital District. The patients/participants provided their written informed consent to participate in this study.

## Author Contributions

JT, NK, TS, LH, and KT: conceptualization. AN, JT, KH, KK, OL, LH, NK, TS, and KT: methodology. AN, JT, OL, NK, TS, and KT: analysis. JT, NK, and KT: supervision. AN: writing—original draft preparation. JT, KH, KK, LH, NK, TS, and KT: writing—review and editing. All authors contributed to the article and approved the submitted version.

## Conflict of Interest

The authors declare that the research was conducted in the absence of any commercial or financial relationships that could be construed as a potential conflict of interest.
