# Supplementary material for: Autoantibodies Against the Immunodominant Bullous Pemphigoid Epitopes Are Rare in Patients With Dermatitis Herpetiformis and Coeliac Disease
Source: Front Immunol. 2020 Sep 25;11:575805. doi: 10.3389/fimmu.2020.575805 (PMC7544946; doi:10.3389/fimmu.2020.575805)
Supplement: Supplementary file 1 [file Table_1.DOCX]

**SUPPLEMENTARY MATERIAL**

**Supplementary table 1. The intensity frequencies of 13 fusion proteins frequencies recognized by sera of patients with dermatitis herpetiformis, coeliac disease, bullous pemphigoid and control sera in immunoblotting**. The recognition of 13 BP180-GST fusion proteins (FP1–13) by the sera of dermatitis herpetiformis (DH), coeliac disease (CD), bullous pemphigoid (BP) and control subjects (Ctrl) in immunoblotting was densitometrically quantified and classified into four categories: 0=no band, 1=weak band, 2=strong band, 3=very strong band.

|  | **Intensity** | **FP1** | **FP2** | **FP3** | **FP4** | **FP5** | **FP6** | **FP7** | **FP8** | **FP9** | **FP10** | **FP11** | **FP12** | **FP13** |
| --- | --- | --- | --- | --- | --- | --- | --- | --- | --- | --- | --- | --- | --- | --- |
| **DH** | 0 | 0 | 1 | 1 | 1 | 20 | 2 | 6 | 1 | 0 | 9 | 12 | 17 | 16 |
|  | 1 | 11 | 12 | 5 | 4 | 0 | 12 | 11 | 11 | 11 | 11 | 7 | 3 | 2 |
|  | 2 | 6 | 7 | 7 | 9 | 0 | 5 | 3 | 4 | 4 | 0 | 1 | 0 | 1 |
|  | 3 | 3 | 0 | 7 | 6 | 0 | 1 | 0 | 4 | 5 | 0 | 0 | 0 | 1 |
|  | Total | 20 | 20 | 20 | 20 | 20 | 20 | 20 | 20 | 20 | 20 | 20 | 20 | 20 |
| **CD** | 0 | 2 | 1 | 1 | 4 | 20 | 5 | 4 | 5 | 3 | 12 | 15 | 19 | 15 |
|  | 1 | 13 | 15 | 10 | 8 | 0 | 6 | 14 | 9 | 10 | 8 | 5 | 1 | 5 |
|  | 2 | 1 | 4 | 6 | 5 | 0 | 5 | 2 | 6 | 7 | 0 | 0 | 0 | 0 |
|  | 3 | 4 | 0 | 3 | 3 | 0 | 4 | 0 | 0 | 0 | 0 | 0 | 0 | 0 |
|  | Total | 20 | 20 | 20 | 20 | 20 | 20 | 20 | 20 | 20 | 20 | 20 | 20 | 20 |
| **BP** | 0 | 4 | 2 | 8 | 1 | 8 | 5 | 12 | 8 | 7 | 11 | 22 | 12 | 11 |
|  | 1 | 9 | 9 | 5 | 12 | 6 | 10 | 8 | 10 | 11 | 8 | 1 | 7 | 9 |
|  | 2 | 2 | 6 | 6 | 5 | 5 | 7 | 2 | 3 | 4 | 2 | 0 | 3 | 1 |
|  | 3 | 8 | 6 | 4 | 5 | 4 | 1 | 1 | 2 | 1 | 2 | 0 | 1 | 2 |
|  | Total | 23 | 23 | 23 | 23 | 23 | 23 | 23 | 23 | 23 | 23 | 23 | 23 | 23 |
| **Ctrl** | 0 | 1 | 1 | 1 | 0 | 21 | 3 | 7 | 2 | 1 | 13 | 11 | 20 | 20 |
|  | 1 | 16 | 18 | 9 | 6 | 0 | 11 | 10 | 10 | 13 | 8 | 9 | 1 | 1 |
|  | 2 | 3 | 2 | 6 | 7 | 0 | 4 | 4 | 6 | 4 | 0 | 1 | 0 | 0 |
|  | 3 | 1 | 0 | 5 | 8 | 0 | 3 | 0 | 3 | 3 | 0 | 0 | 0 | 0 |
|  | Total | 21 | 21 | 21 | 21 | 21 | 21 | 21 | 21 | 21 | 21 | 21 | 21 | 21 |

**Supplementary table 2.** **Dermatitis herpetiformis, coeliac disease, bullous pemphigoid and control sera differ in the recognition of BP180 fusion proteins in epitope mapping.** Fusion proteins, whose recognition shows significant inhomogeneity between bullous pemphigoid, coeliac disease, dermatitis herpetiformis and control group with P values less than .05 are highlighted in bold font. The significance threshold was set at .05.

| **Fusion protein** | **Fisher's exact test** | **Exact 2-sided P value** |
| --- | --- | --- |
| FP1 | **16.029** | **.040** |
| FP2 | **18.345** | **.008** |
| FP3 | 14.010 | .107 |
| FP4 | 12.556 | .151 |
| FP5 | **34.605** | **3.748E-10** |
| FP6 | 7.435 | .602 |
| FP7 | 9.631 | .339 |
| FP8 | 12.440 | .168 |
| FP9 | **16.398** | **.038** |
| FP10 | 8.293 | .381 |
| FP11 | **13.429** | **.011** |
| FP12 | **16.412** | **.006** |
| FP13 | **15.539** | **.015** |

**Supplementary table 3. Sera of patients with dermatitis herpetiformis, coeliac disease, and bullous pemphigoid, and from controls recognizes BP180 epitopes differently.** Epitope mapping data (Supplementary table S1) was analyzed pairwise for each fusion protein (FP) using Fisher’s exact test. Fisher’s exact test values and 2-sided P values are shown. Statistically significant 2-sided P values (P < .05) are highlighted in bold font. BP, bullous pemphigoid; CD, coeliac disease; Ctrl, Control; DH, dermatitis herpetiformis.

|  |  | **FP1** | **FP2** | **FP3** | **FP4** | **FP5** | **FP6** | **FP7** | **FP8** | **FP9** | **FP10** | **FP11** | **FP12** | **FP13** |
| --- | --- | --- | --- | --- | --- | --- | --- | --- | --- | --- | --- | --- | --- | --- |
| **Ctrl**  **vs DH** | test value P value | 3.281 | 1.145 | 0.834 | 6.514 | - | 2.261 | 2.088 | 3.910 | 4.708 | - | 2.627 | - | - |
|  |  | .389 | .697 | .918 | .085 | - | .551 | .368 | .306 | .195 | .999 | .255 | .999 | 0.093 |
| **Ctrl**  **vs DH** | test value  P value | 3.717 | 4.052 | 1.781 | 1.885 | - | 1.384 | 0.340 | 1.057 | 1.652 | - | 0.537 | - | 2.694 |
|  |  | .264 | .098 | .754 | .665 | - | .854 | 0.999 | .896 | .827 | .354 | .871 | .343 | .313 |
| **Ctrl  vs BP** | test value P value | **9.183** | **11.249** | 6.650 | 3.829 | **20.397** | 2.220 | 3.026 | 4.658 | 5.484 | 3.374 | **10.980** | **9.824** | **11.553** |
|  |  | **.020** | **.005** | .087 | .252 | **2.E-06** | .521 | .374 | .201 | .132 | .420 | **.002** | **.006** | **.002** |
| **CD  vs DH** | test value P value | 5.407 | 1.371 | 3.493 | 5.009 | - | 4.860 | 1.034 | 6.816 | **8.337** | - | 1.613 | - | 3.122 |
|  |  | .109 | .731 | .319 | .183 | - | .201 | .651 | .076 | **.025** | .527 | .501 | .605 | .407 |
| **CD  vs BP** | test value P value | 2.880 | **8.173** | 7.089 | 2.799 | **19.584** | 2.830 | 6.405 | 3.154 | 3.138 | 3.149 | - | **9.301** | 4.088 |
|  |  | .472 | **.030** | .066 | .420 | **9.E-06** | .447 | .051 | .361 | .353 | .397 | .081 | **.008** | .175 |
| **DH  vs BP** | test value P value | **7.804** | 6.828 | 6.193 | 5.226 | **19.584** | 1.804 | 3.429 | 6.155 | **9.681** | 3.747 | **8.040** | 5.730 | 5.752 |
|  |  | **.041** | .068 | .105 | .132 | **9.E-06** | .710 | .303 | .109 | **.019** | .262 | **.009** | .060 | .094 |
